# Patterns of Pupillary Activity During Binocular Disparity Resolution

**DOI:** 10.3389/fneur.2018.00990

**Published:** 2018-11-26

**Authors:** Carey D. Balaban, Alex Kiderman, Mikhaylo Szczupak, Robin C. Ashmore, Michael E. Hoffer

**Affiliations:** ^1^Departments of Otolaryngology, Neurobiology, Communication Sciences and Disorders, and Bioengineering, University of Pittsburgh, Pittsburgh, PA, United States; ^2^Neuro Kinetics, Inc., Pittsburgh, PA, United States; ^3^Department of Otolaryngology, University of Miami Hospital, Miami, FL, United States; ^4^Neurological Surgery, University of Miami Hospital, Miami, FL, United States; ^5^Sports Performance and Wellness Institute, University of Miami Hospital, Miami, FL, United States

**Keywords:** convergent eye movements, virtual environment, pupil responses, synkinesias, human

## Abstract

This study examined the dynamic coordination between disconjugate, vergence eye movements, and pupil size in 52 normal subjects during binocular disparity stimulation in a virtual reality display. Eye movements and pupil area were sampled with a video-oculographic system at 100 Hz during performance of two tasks, (1) fusion of a binocular disparity step (±1.5° of visual angle in the horizontal plane) and (2) pursuit of a sinusoidally varying binocular disparity stimulus (0.1 Hz, ±2.6° of visual angle in the horizontal plane). Pupil size data were normalized on the basis of responses to homogeneous illumination increments ranging from 0.42 to 65.4 cd/m^2^. The subjects produced robust vergence eye movements in response to disparity step shifts and high fidelity sinusoidal vergence responses (*R*^2^ relative to stimulus profile: 0.933 ± 0.088), accompanied by changes in pupil area. Trajectory plots of pupil area as a function of vergence angle showed that the pupil area at zero vergence is altered between epochs of linear vergence angle—pupil area relations. Analysis with a modified Gath-Geva clustering algorithm revealed that the dynamic relationship between the ocular vergence angle and pupil size includes two different transient, synkinetic response patterns. The near response pattern, pupil constriction during convergence and pupil dilation during divergence, occurred ~80% of the time across subjects. An opposite, previously undescribed synkinetic pattern was pupil constriction during divergence and pupil dilatation during convergence; it occurred ~15% of the time across subjects. The remainder of the data were epochs of uncorrelated activity. The pupil size intercepts of the synkinetic segments, representing pupil size at initial tropia, had different relationships to vergence angle for the two main coordinated movement types. Hippus-like movements of the pupil could also be accompanied by vergence movements. No pupil coordination was observed during a conjugate pursuit task. In terms of the current dual interaction control model (1), findings suggest that the synkinetic eye and pupillary movements are produced by a dynamic switch of the influence of vergence related information to pupil control, accompanied by a resetting of the pupil aperture size at zero-vergence.

## Introduction

Visual cues for locating three-dimensional objects include binocular disparity, blur, and size change. These cues are used to control disconjugate (convergence and divergence) eye movements that track objects as they vary in depth. Binocular disparity drives an extraocular control process named fusional convergence, while blur-driven eye movements are termed accommodative convergence. These disconjugate eye movements are accompanied by pupil size changes and lens accommodation. For example, when tracking an approaching object, the “near triad” synkinesis (2) is a coordinated execution of convergent eye movements, pupillary constriction (miosis), and increased lens curvature. The opposite response occurs when one tracks a receding object; the eyes will diverge, pupil dilate, and the lens curvature decreases. The dynamic interactions between accommodation and accommodative vergence eye movements have been studied extensively and modeled quantitatively (3–5). This study examines dynamic coupling between vergence eye movements and pupil control during binocular disparity vergence tasks.

Instantaneous pupil size reflects several control signals to sympathetic and parasympathetic preganglionic neurons. The most extensively studied dynamic pupillary control system is the consensual pupillary light response, the adjustments of pupil size for ambient illumination (6–12). The pupillary component of accommodative responses has been termed the “pupillary near reflex” (13, 14). Physiological hippus, a spontaneous fluctuation in pupil diameter at a dominant frequency of ~0.5–0.7 Hz, is produced by variations in central parasympathetic drive (15). Slower pupillary fluctuations related to respiratory cycle control and/or respiratory sinus arrhythmia also appear to be modulated by variations in parasympathetic outflow (16). Finally, factors such as attentional load and task experience can influence pupil size during performance of cognitive tasks (17). These cognitive influences appear to be mediated by a central network that includes descending cortical pathways to the supraoculomotor area and surrounding reticular formation (1, 18, 19).

**Figure 1:**
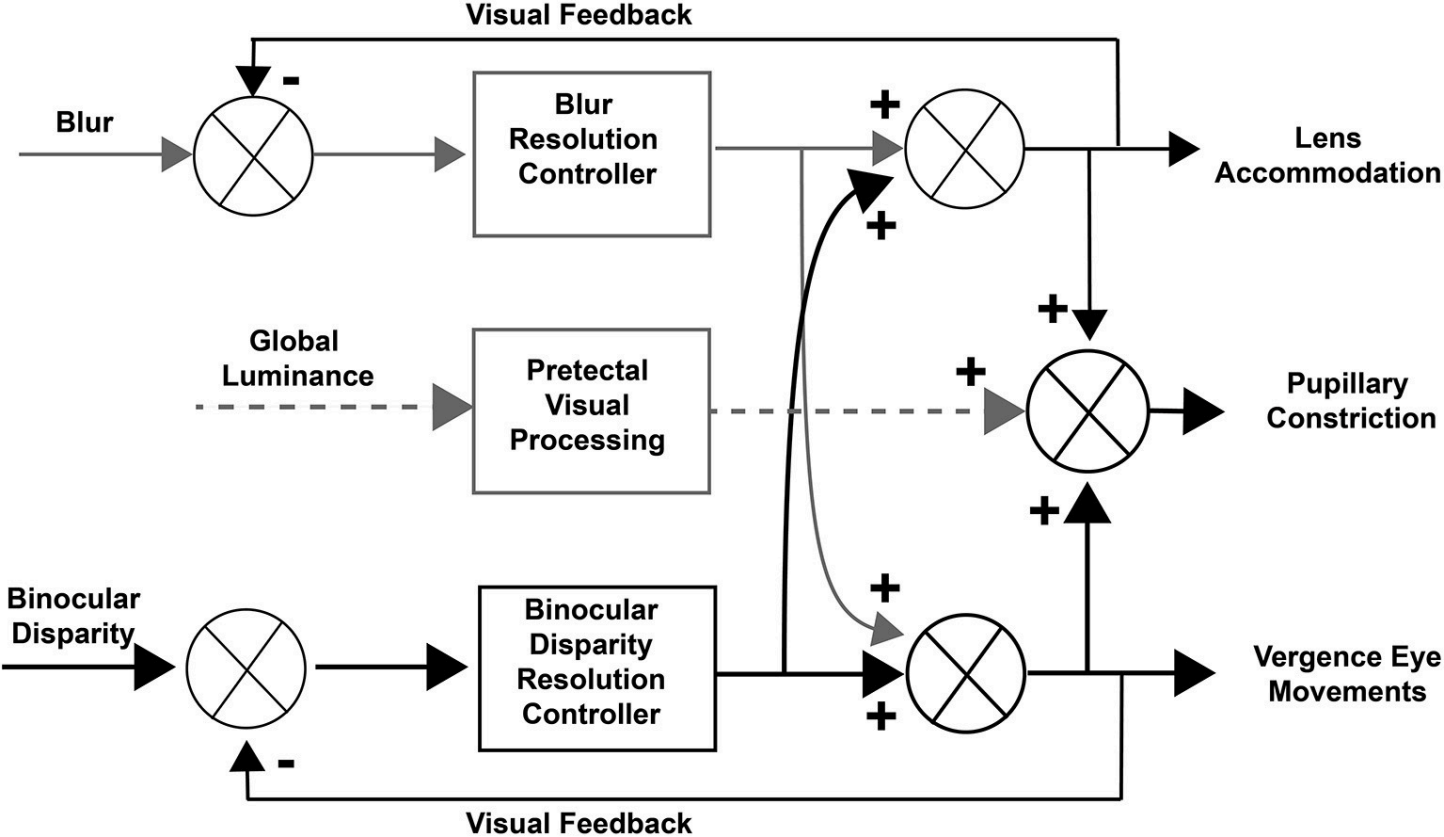
Dual interaction model for coordinated control of vergence eye movements and pupil size. This basic dual interaction model (1) includes separate paths for the influence of blur, binocular disparity, and global illumination on lens curvature, pupil size, and ocular convergence. Blur provides primary control for lens adjustments, binocular disparity drives convergence (to prevent diplopia), and global illumination drives the pupillary light reflex.

Concepts of control of the pupillary component of the near response were summarized recently in a modified dual interaction model by McDougal and Gamlin (1), which posits a central interaction between blur and binocular disparity controllers (3) that is upstream to their individual contributions to pupillary size control. Their model includes a pupillary light reflex pathway, influenced by global luminance pathways through the pretectum (Figure 1). Independent assessment of these subsystems is clearly possible and it would both test and refine these operating models of coordinated extraocular muscle and pupil motor activities.

This study uses a virtual reality display system to introduce binocular disparity alone, with neither blur of the image nor changes in ambient illumination. This selective stimulus allows a comparison of eye movement and pupillary size control during a binocular disparity step task, a binocular disparity pursuit task, and a versional pursuit task at the same frequency. The model predictions are then analyzed on two scales. A macroscale analysis examines vergence eye movement response as the product of a simple transfer function model for the binocular disparity resolution controller. The coupled component of the pupil response is represented by a transfer function from the literature (9), which assumes that pupil size at zero vergence remains invariant. A microscale analysis, on the other hand, uses time series approaches to identify epochs of synkinetic relationships between vergence eye movements and pupil area, which include gain differences and changes in both the range and the center of the range for pupil area regulation. These latter analyses demonstrate that the coordination of extraocular muscle-driven vergence and pupillary area during dynamic vergence pursuit differ from the features revealed by the traditional step testing (steady state measurements at two fixation points or binocular disparities). More significantly, the disparity-induced convergence pursuit task revealed previously undescribed, synkinetic patterns of dynamic coordination of pupillary and extraocular muscle responses.

## Materials and methods

Control subjects were recruited at Madigan Army Medical Center (35 subjects: 26 males, 9 females) and the University of Miami (17 subjects: 10 males, 7 females). Subjects ranged in age from 21 to 45 years [mean 28.7 ± 6.3 (S.D.) years]. Informed consent was obtained. Control subjects were selected who had no history of otologic or ophthalmologic disorders, and no history of traumatic brain injury or neurologic disorders. In addition, these control subjects were not taking any prescription or over the counter medicines that would impair or affect vestibular function or performance on the test battery. The project was approved by the IRBs at the University of Miami, Madigan Army Medical Center, and the University of Pittsburgh.

The experimental apparatus used in this study was a portable 3D head mounted display (HMD) system with integrated clinical eye tracking technology (I—PAS ™; I-Portal® Portable Assessment System, Neuro Kinetics, Inc., Pittsburgh, PA, USA). Within this device, each eye views an independent circular segment of a 1,920 × 1,080 pixel stimulus display that subtends a 60° diagonal field of view. The device has integral video-based eye tracking, performed under continuous 940 nm infrared illumination at a sampling rate of 100 Hz. Pupils are detected by identification of luminance boundaries. The pupil area is measured for each image, and eye position is calculated from the centroid of the identified pupil. Horizontal (±30° range) and vertical (±20° range) eye tracking spatial resolution is on the order of 0.02°, while spatial resolution for torsional eye movement (±10° range) is < 0.1°. Subjects can also adjust the focus of the video image across a 6 diopter range.

Neuro Kinetics VEST™ software was used to run the battery of tests and for data collection. All stimuli were rendered in the virtual environment that was created by the enclosed video display, and stimulus refresh rates were synchronized with the eye tracking sampling rate. Eye movement recordings were calibrated for a series of conjugate horizontal and vertical gaze shifts, using spot targets subtending ~0.1° of visual angle. For calibrating the pupillary light reflex, the subjects viewed a 5° (visual angle) disc centered on the visible area of each screen half, illuminated at intensities ranging from 0.42 to 65.4 cd/m^2^. For analysis of responses during vergence tasks, the pupil area (A_raw_) was normalized for each subject based on pupillary light reflex responses. The maximum (A_max_) and minimum (A_min_) pupil areas were determined separately for left and right eyes for responses to low (0.42 cd/m^2^) and high (65.4 cd/m^2^) brightness stimulus. The normalized values were calculated for each eye as A_norm_ = 100^*^(A_raw_−A_min_)/(A_max_−A_min_); the instantaneous mean of the left and right pupil values was used for identification of synkinetic oculomotor and pupil response components.

**Figure 2:**
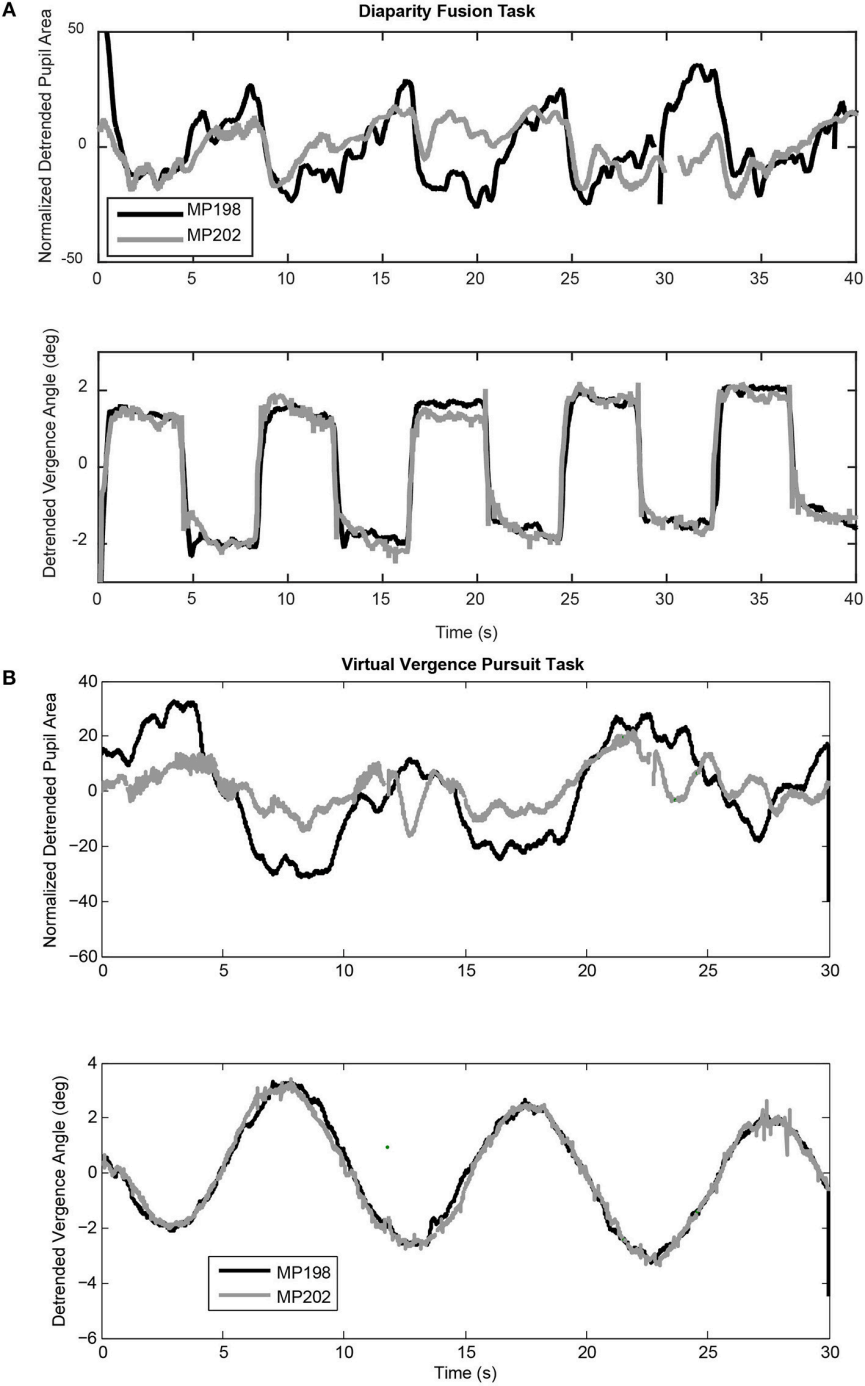
Examples of coordinated pupil and eye movements during the virtual binocular disparity vergence task. **(A)** Detrended data from two subjects (MP198 and MP202) during a step binocular disparity fusion task. The upper panel shows the normalized pupil area traces and the lower panel shows the ocular vergence angle relative to the tropia at calibration. **(B)** Detrended data from the same subjects for a binocular disparity tracking task at 0.1 Hz. Note the highly consistent vergence eye movements for both the binocular disparity step tasks and the disparity tracking tasks of both subjects. Data from MP198 before detrending are shown in the supplemental data section (Figure S1).

Targets for the disparity fusion (“vergence tracking”) task were a white square with red center that covered ~ 0.1° visual angle of each eye. The total field luminance during presentation of the square, measured with a spot luminance detector incorporating a LDM-9901 sensor (Gigahertz-Optik, Germany), ranged from 0.05 to 0.06 cd/ m^2^. The vergence disparity step task began with the illuminated targets at a central fixation position for each eye. The targets were then shifted at 4 s intervals between a disparity requiring a 1.5° convergence and a disparity requiring a 1.5° divergence in order to achieve binocular fusion. Five cycles of alternating convergence and divergence were presented over a 40 s duration (Figure 2A). For the vergence pursuit (tracking) task, the trial began with illumination of the two monocular targets at the initial focal point phoria (equivalent to ~1 m in virtual depth). The target then moved smoothly through 3 cycles of a sinusoidal profile, such that the monocular targets moved simultaneously laterally and then medially to produce binocular disparity (i.e., the left eye target moved leftward while the right eye target moved rightward, then the left eye target moved rightward while the right eye target moved leftward) with a cycle duration of 10 s. During this sinusoidal movement, the maximum deviation of the response from the initial position was ±2.6° of visual angle in the horizontal plane. Since there is no stimulus blur introduced, the accommodation produced by this vergence angle is expected to be on the order of 0.5 diopter and to be linear with visual angle (20). For the versional smooth pursuit task, the stimuli monocular spots were moved sinusoidally to the left and then to the right (±10° excursion) with a cycle duration of 10 s.

The calibrated data were exported as Excel files and analyzed with MATLAB (MathWorks, Natick, MA) and SPSS Statistics 24 (IBM). The eye movements in the disparity step task were modeled as the weighted sum of first order high and low pass representations of the vergence target position with a processing delay. Nonlinear least squares regression (“lsqnonlin.m” function in MATLAB) was used to estimate parameters for the vergence disparity response as a weighted sum of phasic ($\frac{K_{vh}se^{-t_{v}s}}{s+1}$) and tonic ($\frac{K_{vl}e^{-t_{v}s}}{0.25s+1}$) processes, with delay *t*_v_ and gains *K*_vh_ (phasic process) and *K*_vl_, (tonic process), respectively. The delay parameter accounts for the reaction time to the binocular disparity step stimulus; it was set at zero for the binocular disparity pursuit task. Based upon Sun et al. (9), the pupil dynamics were fitted from the vergence data by a transfer function for pupil motion, $\frac{K_{p}e^{-tps}}{0.28s+1}$, with delay *t*_p_ and gain *K*_p_, which estimates the near response sensitivity directly. Symmetry was tested by fitting separate gains for half-cycles of convergence vs. divergence and for half-cycles of pupil constriction versus dilatation.

## Results

### General observations

Both binocular fusion stimuli produced robust, high fidelity convergent, and divergent eye movements. The binocular disparity step stimuli produced an alternating sequence of rapid converging and diverging movements to fuse the disparate targets (Figure 2A, lower trace). The binocular disparity pursuit stimulus (Figure 2B) also produced a robust tracking sequence of divergent and convergent eye movements. These eye movements are a primary response to binocular disparity. They are accompanied by pupillary responses that vary during eye movements and between subjects. The deterministic properties and variability in the relationship between the pupillary responses and eye movements will now be examined for each disparity task.

The findings are described sequentially from the perspectives of macroscale and microscale behavior. Metrics of macroscale behavior were derived by (1) analysis of responses relative to the stimulus profile, and (2) analyses based upon a lumped parameter linear systems approach for eye and pupil movements from an entire trial. These approaches are presented initially for the binocular disparity step and binocular disparity pursuit tasks. These macroscale approaches assume that both the range and the center of the range for pupil area regulation are stationary during a measurement trial. The ensuing microscale perspective uses bivariate analyses of the coordinated trajectories of eye and pupil movements to characterize independent and synkinetic control epochs. This microscale analysis explicitly characterizes time-dependent behavior of the pupil regulatory range and its relationship to vergence angle regulation.

### Macroscale analysis approach

#### Binocular disparity step

The binocular disparity step stimulus sequence produced alternating convergent and divergent eye movements, accompanied by a more variable modulation of pupil area (Figure 2A). Table 1A shows average measurements of the pupil area and the vergence angle of the eyes during the steady state of the convergence and divergence fusion responses; the ratio of these measures has been used in the literature to provide an estimate of the static (or steady-state) sensitivity of the pupil “near response.” The oculomotor vergence responses were symmetric for converging and diverging disparity fusion movements. In contrast to the symmetric oculomotor vergence behavior, the average magnitude of pupillary area changes was significantly greater in the diverging direction than the converging direction (Table 1A, paired *t*-test, *t*_(49)_ = 6.25, *p* < 0.01). The near response magnitudes, estimated for constriction in the converged, and dilation during diverged eye movements, were also significantly greater in the diverged direction (*p* < 0.01). The lumped peak-to-peak estimate of the near response magnitude was 6.21 ± 0.60 % area (relative to light reflex range)/degree for constriction during convergence (relative to resting tropia) or dilation during divergence.

Table 1Disparity step task responses.

**Table:** 

**Component**	**Direction**	**Average magnitude or *Gain* (±SE)**	**Near response sensitivity**
**A. MEASUREMENTS FROM STEADY-STATE EPOCHS (*****n*** = **52 SUBJECTS)**			
Vergence	Toward midline (convergent disparity)	1.40 ± 0.07° *0.93 ± 0.05*
	Away (divergent disparity)	1.44 ± 0.07° *0.96 ± 0.05*
Pupil	Toward midline	7.60 ± 0.89%** (Normalized re: light response)	−5.39 ± 0.52 %/° convergence**
	Away	10.00 ± 0.88% (Normalized re: light response)	−7.13 ± 0.60 %/° convergence
**p < 0.01 relative to opposite direction.

**Table:** 

**Component**	**Direction**	**Magnitude or** ***Gain*** ± **S.E**.	**Comment**
**B. MODEL PARAMETERS (*****n*** = **52 SUBJECTS)**			
High pass vergence magnitude	Converge	0.173 ± 0.045 *0.108 ± 0.017*	*Fully Rectified and Symmetric*
	Diverge	−0.169 ± 0.035 *−0.105 ± 0.013*
Low pass vergence magnitude	Converge	1.409 ± 0.069 *0.88 ± 0.04*	*Symmetric*
	Diverge	1.485 ± 0.071 *0.93 ± 0.04*
Pupil (re: vergence; “near response gain”)	Constrict	−5.032 ± 0.610 % / °convergence	*Asymmetric (p < 0.001)*
	Dilate	−7.983 ± 0.595 %/ °convergence

*Gains, shown in italic font below the response magnitudes, were obtained by dividing magnitudes by the virtual stimulus magnitude in that direction, 2.6°*.

The impression that the converging eye movement responses were brisker than the diverging responses (Figure 2A, lower traces) was tested by linear systems modeling of the eye movement responses as the sum of high and low pass representations of the vergence target position (see Methods). The goodness of fit of the model to the vergence eye movements was very robust, with average coefficients of determination (*R*^2^) of 0.84 ± 0.03. The estimated processing delays (t_v_) were 0.26 ± 0.02 s and the estimated gains are listed in Table 1B. The high pass gain values were rectified, but of the same magnitude for shifts of the stimuli in either nasal or temporal directions; hence, there was a phasic convergence when disparity changed abruptly. The low pass magnitudes for the convergent and divergent eye movements were symmetric and did not differ from the static responses estimates in Table 1A.

The contribution of pupillary motion dynamics to this directional asymmetry was tested by modeling pupil motion as a function of the drive that produces the vergence eye movements (9). The gain estimate from this model represents the responsiveness of normalized pupil diameter per degree of vergence (gain of the pupil “near response”). The model from the literature explained roughly 30% of the variance in the pupil traces as a function of only the vergence behavior (*R*^2^ = 0.28 ± 0.03) and the estimate of the delay parameter (0.19 ± 0.02 s) did not differ significantly from the 0.2 s delay estimate for the pupillary motor reaction during the light response (9). Consistent with the outcome of the steady state analysis (Table 1A), the average sensitivity (% area/degree convergence) when diverging was significantly greater than the sensitivity converging [paired *t*_(49)_ = 6.97, *p* < 0.001].

#### Sinusoidal tracking task

**Figure 3:**
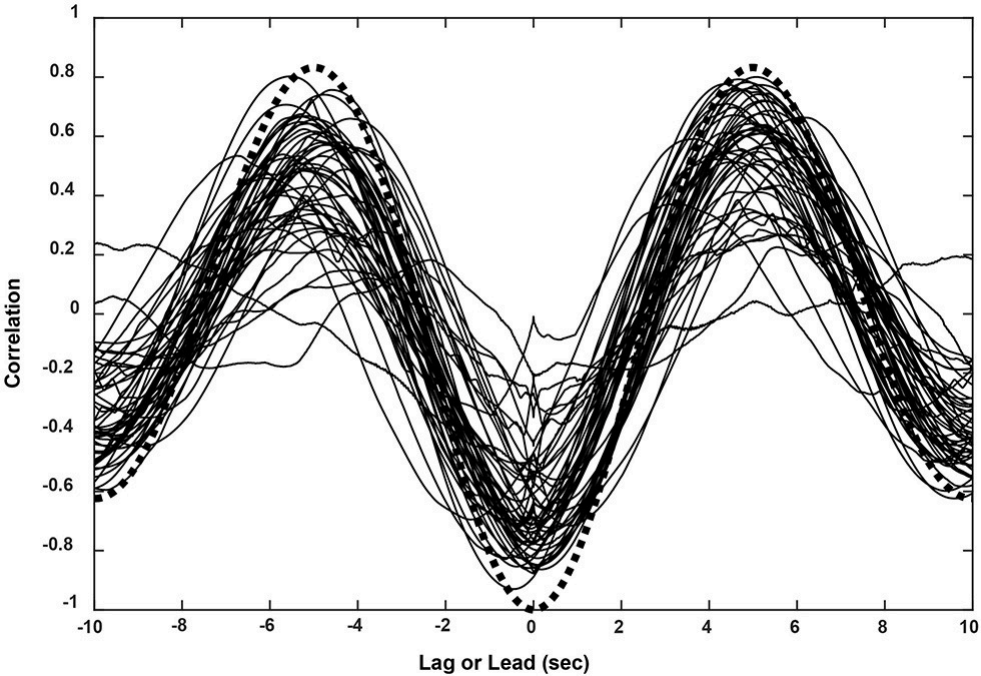
Cross-correlation functions for eye movement and pupil responses (sinusoidal pursuit task) from all 52 subjects. The functions corresponded closely to the theoretical result for two cosines at the stimulus frequency of 0.1 Hz (black dashed line).

The subjects' tracking of the virtual stimuli (disparity simulation varying sinusoidally from 2.6° divergence to 2.6° convergence at 0.1 Hz) produced symmetric smooth convergence eye movements, accompanied by consensual pupillary area changes. Examples of the average vergence angle and average pupillary area (normalized to the light reflex response) are shown in Figure 2B. The initial divergent eye movement tracking was accompanied by pupillary dilation, followed by convergent eye tracking movements that were accompanied primarily by pupillary constriction. The modulation amplitudes of the eye movement [paired *t*_(51)_ = 4.37, *p* < 0.001] and of the pupil area [paired *t*_(51)_ = 5.19, *p* < 0.001] were both greater pursuing virtual targets toward the midline, which is a convergence response, than during a divergence response (Table 2A). The eye movements displayed extremely high fidelity to a sinusoidal tracking profile. The modulation in the convergence direction did not differ significantly from the intended ± 2.6° vergence modulation, but the divergence response was smaller. There was a small linear drift of −0.011± 0.002 (SE) deg/s in the center of modulation toward divergence, which is equivalent to a gradual divergence (relative to initial tropia) by ~ 0.33° over the 30 s task [*t*_(51)_ = −4.64, *p* < 0.01]. The pupil size was modulated out of phase with the vergence angle (average difference: 167.6 ± 2.5° or 2.925 ± 0.043 rad), but there was no significant linear drift in pupil size during the 30 s task [t_(51)_ = −0.671, *p* > 0.5]. Cross-correlation functions for the detrended subject data (Figure 3) showed the configuration for the correlation of two out-of-phase sine waves (dashed black line) confirming that the virtual stimulus elicited coordinated performance of oculomotor and pupillary components of the near triad.

Table 2Sinusoidal vergence pursuit modulation parameters for eye movements and pupil size.

**Table:** 

**Component**	**Direction**	**Magnitude or *Gain* [±SE]**	**Phase angle re: Stimulus (±SE)**	**R^2^ (±SE)**
**A. SINUSOIDAL ANALYSIS**				
Vergence	Toward midline	2.54 ± 0.11° *0.98 ± 0.04*	172.75 ± 1.26° (3.015 ± 0.022 rad)	0.933 ± 0.088
	Away	2.26 ± 0.10° *0.87 ± 0.04*	
Pupil	Toward midline	23.54 ± 1.57% (Normalized re: light response)	−8.37 ± 4.87° (−0.146 ± 0.085 rad)	0.563 ± 0.198
	Away	13.43 ± 1.96% (Normalized re: light response)

**Table:** 

**Component**	**Direction**	**Magnitude or** ***Gain*** **[**± **SE]**	**Comment**
**B. MODEL PARAMETERS**			
High pass vergence magnitude	Both (rectified)	0.255 ± 0.084 *0.098 ± 0.032*
Low pass vergence magnitude	Converge	2.422 ± 0.128 *0.93 ± 0.05*	*Symmetric*
	Diverge	2.246 ± 0.127 *0.86 ± 0.05*
Pupil(re: vergence; “near response gain”)	Constrict	−7.841 ± 0.727 %/° convergence	*Asymmetric (paired t, t = 2.8, p < 0.01)*
	Dilate	−8.413 ± 0.646 %/° convergence^**^

*Gains, shown in italic font below the response magnitudes, were obtained by dividing magnitudes by the virtual stimulus magnitude in that direction, 2.6°*.

** *p < 0.01, paired t-test*.

Linear systems modeling of the binocular disparity pursuit eye movement responses as the sum of high and low pass representations of the vergence target position was conducted to test the hypothesis that the same basic model can characterize the response dynamics for both binocular disparity step and pursuit responses. Based upon the results for the binocular disparity step response (Table 1B), a single sensitivity parameter was used to characterize a fully rectified high pass component (Table 2B). Repeated measures ANOVA indicated no significant differences in gain estimates for either high or low pass components of the vergence eye movements from the step versus pursuit tasks. In contrast to the results of the binocular disparity step task (which has higher frequency components), the estimates of the pupil near response sensitivity (%/° convergence) for this single, low frequency pursuit task did not differ for converging and diverging movements during the pursuit task. The steady-state sensitivity of the pupillary near response during sinusoidal vergence tracking was estimated directly from the data by (a) the lagged slope (from autoregression analysis) of the bivariate relationship for pupil area as a function of vergence angle (−7.97 ± 0.62 % area/° vergence) and (b) the ratio of the average peak modulations of the pupil area and vergence movements (−8.22 ± 0.62 % area/° vergence). The linear modeling approach was also used to estimate the dynamic near response sensitivity from the vergence pursuit response, using the transfer function $\frac{K_{p}e^{-tps}}{0.28s+1}$. The delay parameter *t*_p_ was set at 0.19 s, the average value from the binocular disparity step response analysis (above). The model could account for approximately half of the variance in the pupil traces (*R*^2^ = 0.51 ± 0.03 S.E.). The near response sensitivity, estimated for the converging (−7.84 ± 0.73 % area/° convergence) and diverging (−8.41 ± 0.65 % area/° convergence) directions, were significantly greater in the diverged direction (paired *t*-test, *p* < 0.01). The model-based, average near response sensitivity estimate for the pursuit task (−8.13 ± 0.75 % area/° convergence) was greater (paired *t*-test, *p* < 0.05) than for the step task (−6.51 ± 0.56 % area/° convergence).

These findings are consistent with the prevailing concept (1, 3) that there is a central coupling between binocular disparity processing and pupillary control. However, the variations in the estimates of the magnitude of this “near response” component in pupillary control provided motivation for a more detailed analysis of the dynamic coordination of pupil and vergence eye movement control during the disparity tracking task.

### Microscale analysis approach

#### Binocular disparity step eye-pupil trajectories

**Figure 4:**
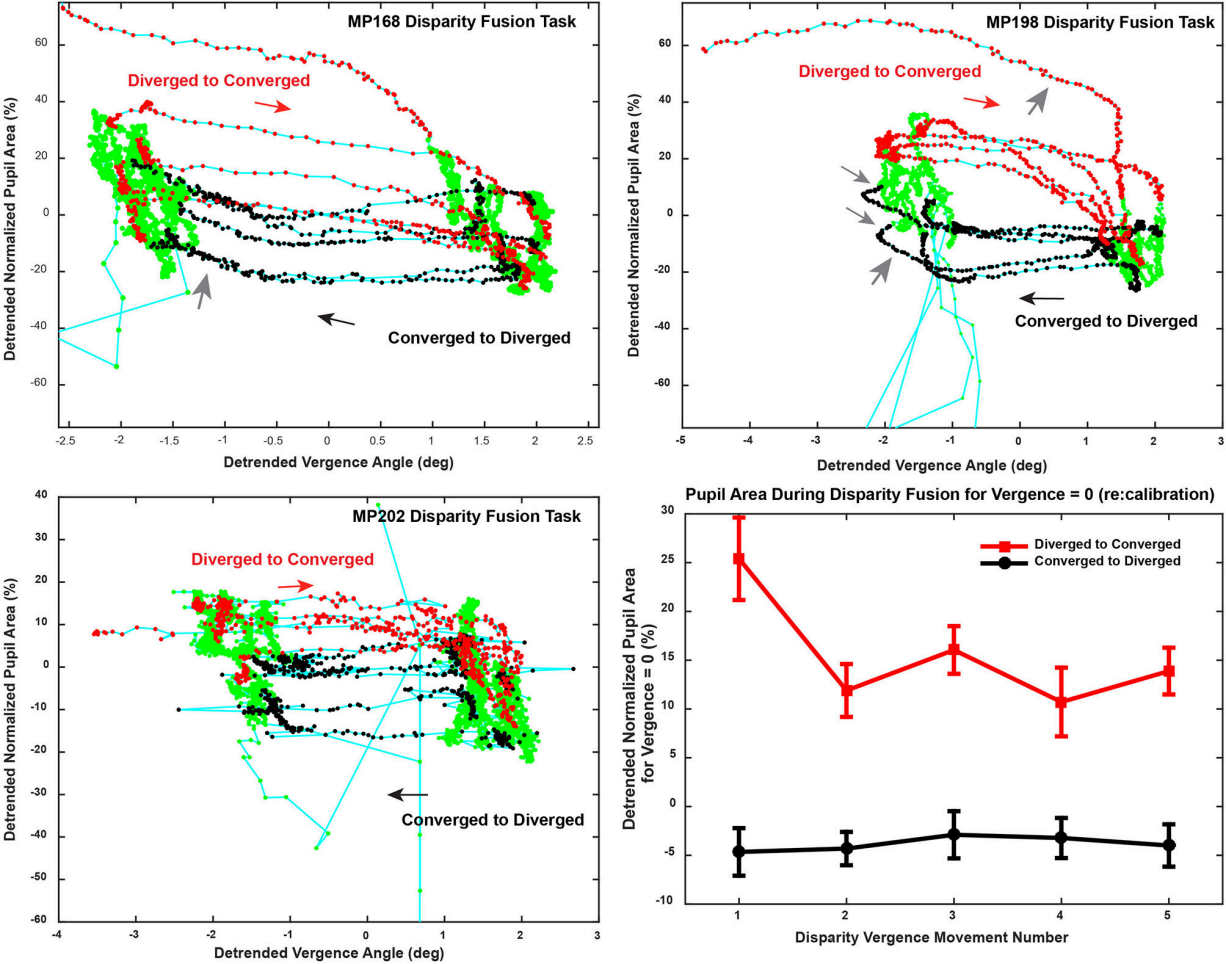
Trajectory of the vergence angle and pupil area during the binocular fusion step task is shown in the upper and lower left panels. The upper right (MP198) and lower left (MP202) panels area are plotted from data shown in in Figure 2A. Successive sample points (100 Hz sample rate) are plotted in green, with the dots indicating the convergent (red dots) and divergent (black dots) disparity-driven responses. The directions of the trajectories between fixations is indicated with arrows. Note that response patterns include periods when there are (1) almost exclusively vergence eye movements (horizontally oriented segments), (2) almost exclusively pupil area changes (vertically oriented segments), (3) combinations that reflect a near response pattern (pupil constriction with convergence and dilation with divergence, larger gray arrows) and (3) a pattern that is opposite the near response (smaller gray arrows). The lower right panel shows the average pupil area (normalized to light reflex range) at zero vergence for successive increments) to show the stability across repeated binocular disparity steps.

The magnitude of the pupillary responses varied on a movement-by-movement basis during reproducible vergence eye movements (e.g., Figure 2A). The variability between epochs of dynamic pupillary area changes across a consistent pattern of vergence eye movements is shown in trajectory plots of normalized pupil size as a function of vergence angle (Figure 4). Successive sample points (100 Hz sample rate) are shown for convergent (red dots) and divergent (black dots) disparity-driven responses. Relationships during steady-state fusion are represented by green dots; cyan lines show the connected trajectory. The representative cases have several noteworthy features. Firstly, a wide range of instantaneous pupillary areas were observed at the convergent and divergent fusion targets during the task. Secondly, the pupillary areas at the initial tropia (represented as zero detrended vergence) tended to differ during the convergent versus the divergent eye movements [Repeated Measures ANOVA, main effect of direction, *F*_(1, 36)_ = 82.45, *p* < 0.001], which indicates that the set point for pupillary control relative to vergence angle is changing on a movement-by-movement basis during the fixation/fusion periods of the task (green dots). Finally, the pupillary component of individual disparity responses shows three patterns relative to the consistent pattern of vergence eye movements (Figure 4): epochs of (1) almost exclusively eye movement (“horizontal segments”) or pupil area changes (“vertical segments”), (2) combinations that reflect a near response pattern (pupil constriction with convergence and dilation with divergence, larger gray arrows) and (3) a pattern that is opposite the near response (e.g., some MP198 data segments in Figure 4 show dilation with convergence, small gray arrows).

#### Sinusoidal pursuit task eye-pupil trajectories

**Figure 5:**
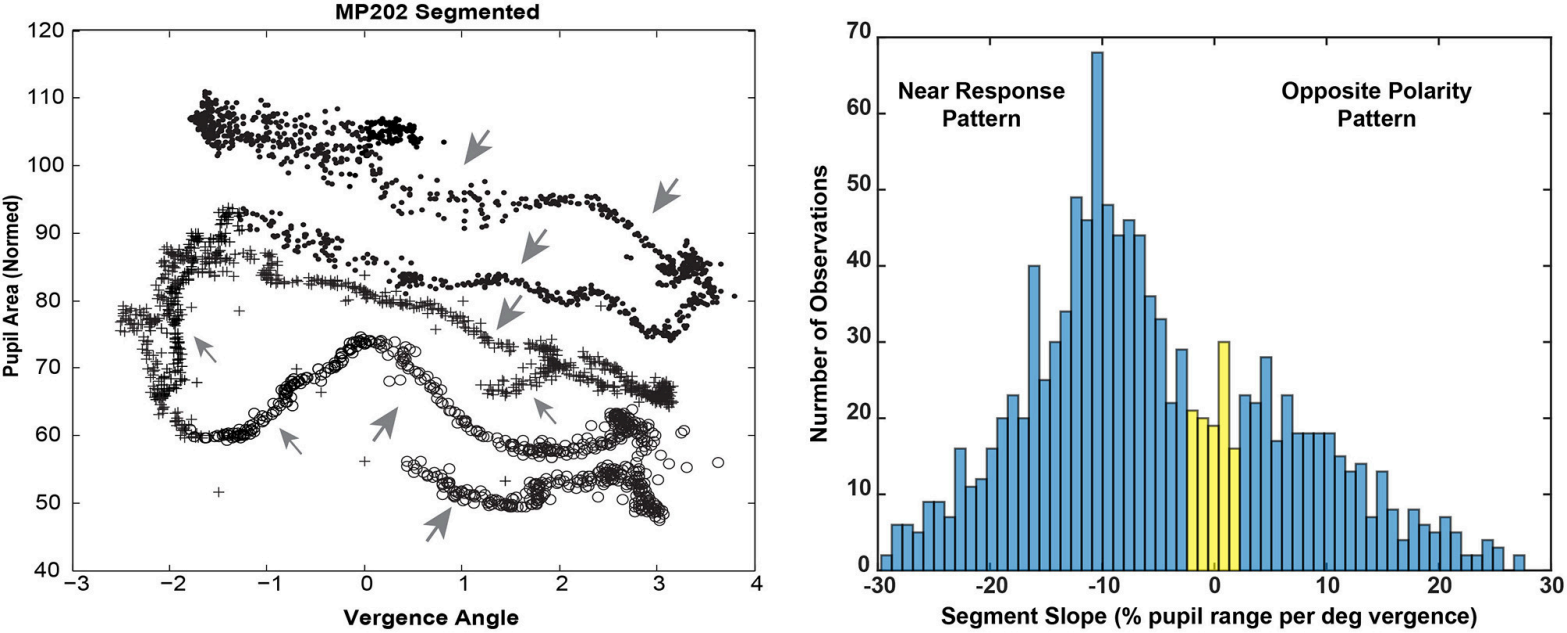
The left panel shows the trajectory of the vergence angle and pupil area for one of the traces shown during the binocular disparity pursuit task in Figure 2B. Note that the graph appears as a series of parallel quasi-linear segments, offset by differences in a pupil size set-point. The right panel shows the bimodal distribution of linear segment slopes. A fuzzy c-means clustering algorithm [(22), MATLAB routine “fcm.m”] indicated that the boundary between two clusters, with at least 0.65 fuzzy class membership (shown in blue), was demarcated by cutoffs of ≤ −2% pupil area range /deg vergence (“near response” pattern) and ≥2% pupil area range /deg vergence (opposite pattern). The intermediate observations (flat slope) are shown in yellow.

Bivariate plots of detrended pupil areas as a function of the detrended disjunctive eye movement measurements showed extensive epochs of linear coupling during the virtual vergence tracking task. Data from a representative subject is shown in Figure 5 (left panel). Like the disparity fusion task plots in Figure 4, each subject showed epochs with different quasilinear associations between concurrent pupil areas and vergence angles, including segments with a near response pattern (pupil constriction with convergence and dilation with divergence, larger gray arrows) and segments with an opposite movement pattern, dilation while converging (smaller gray arrows). Each bivariate plot is dominated typically by a series of parallel segments reflecting the pupillary constriction during convergence (near response pattern), offset by differences in a pupil size set-point. Because the sampled detrended normalized pupil area and detrended vergence angles from each session are a bivariate time series, an analysis technique was applied to objectively identify segments with a homogeneous linear slope. This slope provides an empirical estimate of the influence of the hypothetical disparity control mechanism [McDoughal and Gamlin (1)] on pupillary size. A modified Gath-Geva clustering algorithm (21) was used for objective fuzzy segmentation of the time series into 15 segments with homogeneous properties, based upon preliminary analyses indicating that results were unaffected by more granular divisions of the data into more segments. This published algorithm first applies a principal component decomposition to identify a component that represents the instantaneous pupillary area relative to instantaneous vergence angle. It then applies a clustering algorithm to decompose the data into linear segments, based upon measured homogeneity of the segments, and the fuzzy sets that are used to represent the segments in time.

**Figure 6:**
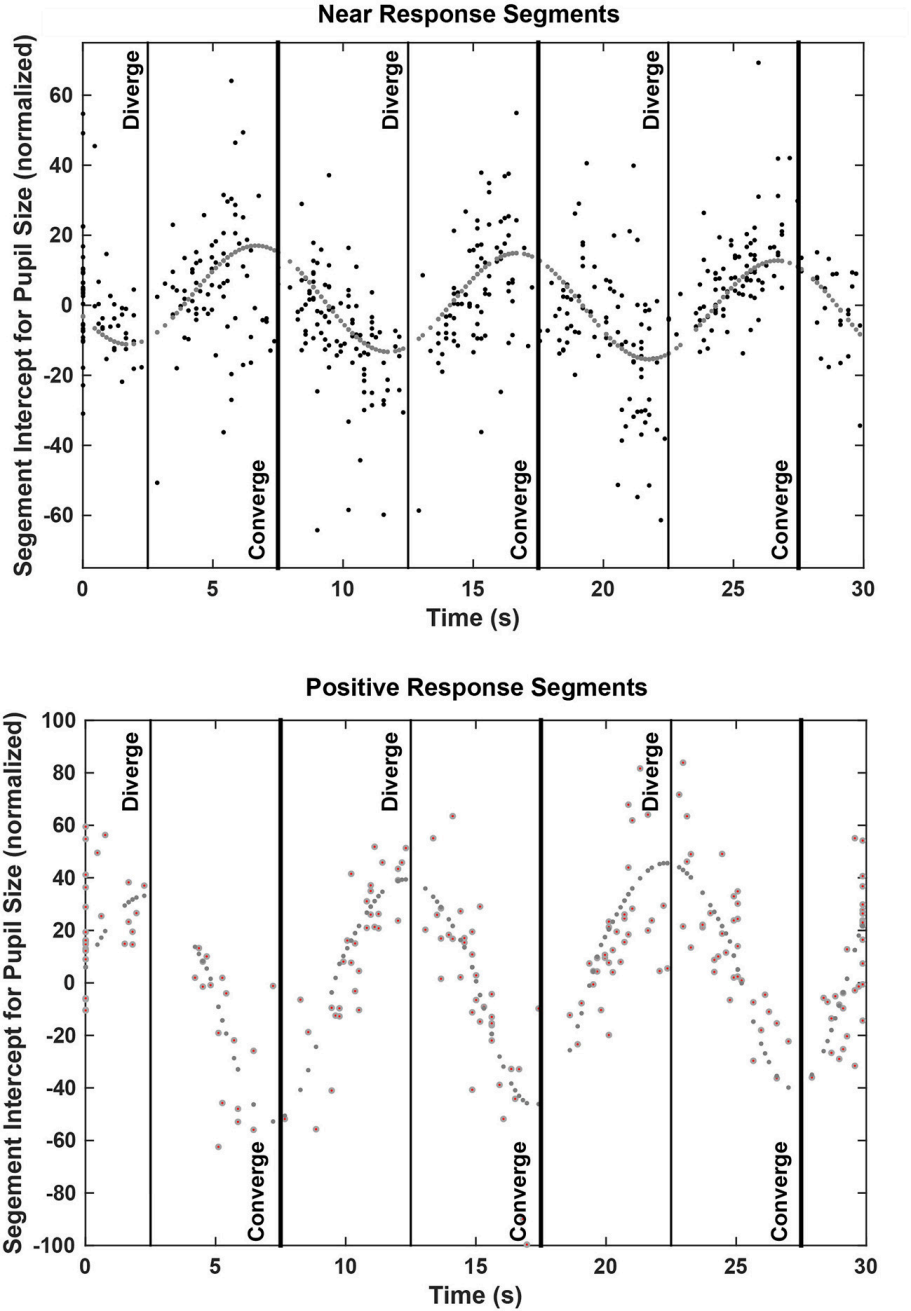
Plots of the pupil size intercepts across all subjects for linear segments of coordinated eye movement and pupil vergence accommodation patterns. The segment intercept is plotted at the onset of the segment; the stimulus times for maximum divergence and convergence are shown by vertical lines.

The distribution of the slopes of linear segments had relative peaks in both the negative and positive slope directions (Figure 5, right panel). A fuzzy c-means clustering algorithm [(22), MATLAB routine “fcm.m”] indicated that the boundary between two clusters, with at least 0.65 fuzzy class membership, was demarcated by cutoffs of ≤ −2% pupil area range /deg vergence (“near response” pattern) and ≥2% pupil area range /deg vergence (opposite pattern). The remaining segments with >0.35 to < 0.65 membership in either class were considered to have a “flat” slope (yellow in Figure 6, right panel). Applying these boundaries across all subjects, 67% of the segments (range: 7–15 segments/subject, average: 10) showed the near response type (slope ≤ −2% pupil area range /deg vergence, average *R*^2^ = 0.670 ± 0.015) and 27% (range: 0–8 segments/subject, average: 4) of the segments showed the opposite relationship (slope ≥ 2% pupil area range /deg vergence, average *R*^2^ = 0.651 ± 0.024). The flat segments [6% of segments across subjects (range: 0–4 segments/subject), yellow in Figure 5, right panel] had a significantly lower, but still reasonably strong *R*^2^ value (0.421 ± 0.052). The near response-type segments were of significantly longer duration (2.39 ± 0.06 s, Tukey HSD tests, *p* < 0.01) than either the segments with opposite polarity (1.17 ± 0.09 s) or the flat (absolute slope < 2% pupil area range/deg vergence) slope segments (1.38 ± 0.19 s), which did not differ from each other. Slope and duration of segments were uncorrelated. Hence, the near response pattern was present 82.8 ± 1.5% (mean ± SE) of the time during vergence trials for individual subjects, while the opposite pattern was present ~16% of the time.

The dominant “near response” pattern, a negative linear relationship between the vergence angle and pupil diameter (slope ≤ −2 % of pupil area range per degree of vergence) consisted of segments with an average slope of 13.3 ± 0.6 % of pupil area range per degree of vergence (mean ± SE).The high coefficient of determination (*R*^2^ = 0.670 ± 0.015) suggests that binocular disparity is a prominent drive during these segments for pupillary constriction during convergence and pupillary dilation during divergence. The magnitudes of the slopes of these segments were significantly greater than the steady state estimates of near response sensitivity and the near response sensitivity estimate from the disparity fusion task in each subject, as shown by repeated measures analysis of variance [*F*_(3, 147)_ = 38.71, *p* < 0.001] followed by pairwise comparisons (*p* < 0.001 for each case).

**Figure 7:**
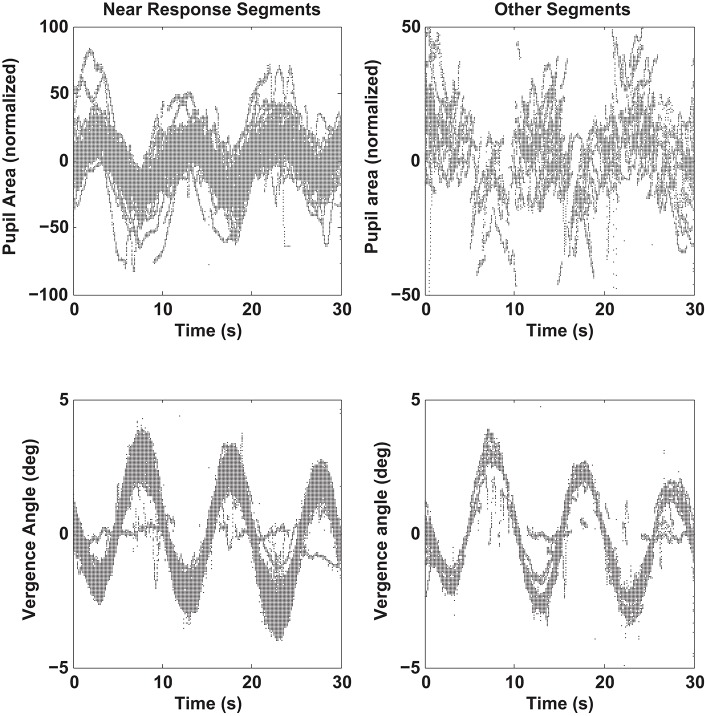
Pupil size recordings (upper graphs) and vergence angle recordings (lower graph) are shown for data segments showing a negative linear relationship between pupil area and vergence angle (“Near Response Segments,” left side) and for segments showing either a positive linear relationship between pupil area and vergence angle or a flat relationship (“Other Response Segments,” left side). Segments of detrended data from all subjects are superimposed, including one outlier subject with little vergence tracking. Note the out-of-phase modulation of the pupil and vergence traces for the “Near Response Segments”.

The zero vergence intercept of the pupil-area for these linear segments estimates the set point of pupil area for each segment, relative to the initial tropia during calibration (defined arbitrarily as 0°). This new set point is likely to reflect other signals for the pupil control, including “aftereffects” of disparity that alter vergence phoria (3) and effects related to cognitive processing load. The intercepts varied with the binocular disparity at the start of the segment for both the near response (negative slope) and positive slope segments (Figure 6). Nonlinear regression was used to model the pupil set points (zero-vergence angle intercepts) as the sum of a constant offset, a linear trend with time and an asymmetric sinusoidal modulation (Table 2). For near response segments, the set point tended to dilate while the target disparity is converged, and to constrict while the target disparity is diverged. Approximately 24% of the variance in the set point of the near response linear segments reflects a constant dilation of almost 4% of light response range and symmetric modulation of about 14.6% of light response range. The pupil size and vergence angle traces from these “near response segments” are extracted and superimposed on the left side of Figure 7. Although the pupil set point changes produce variability, these pupil response segments show smooth, continuous modulation during vergence eye movements.

**Figure 8:**
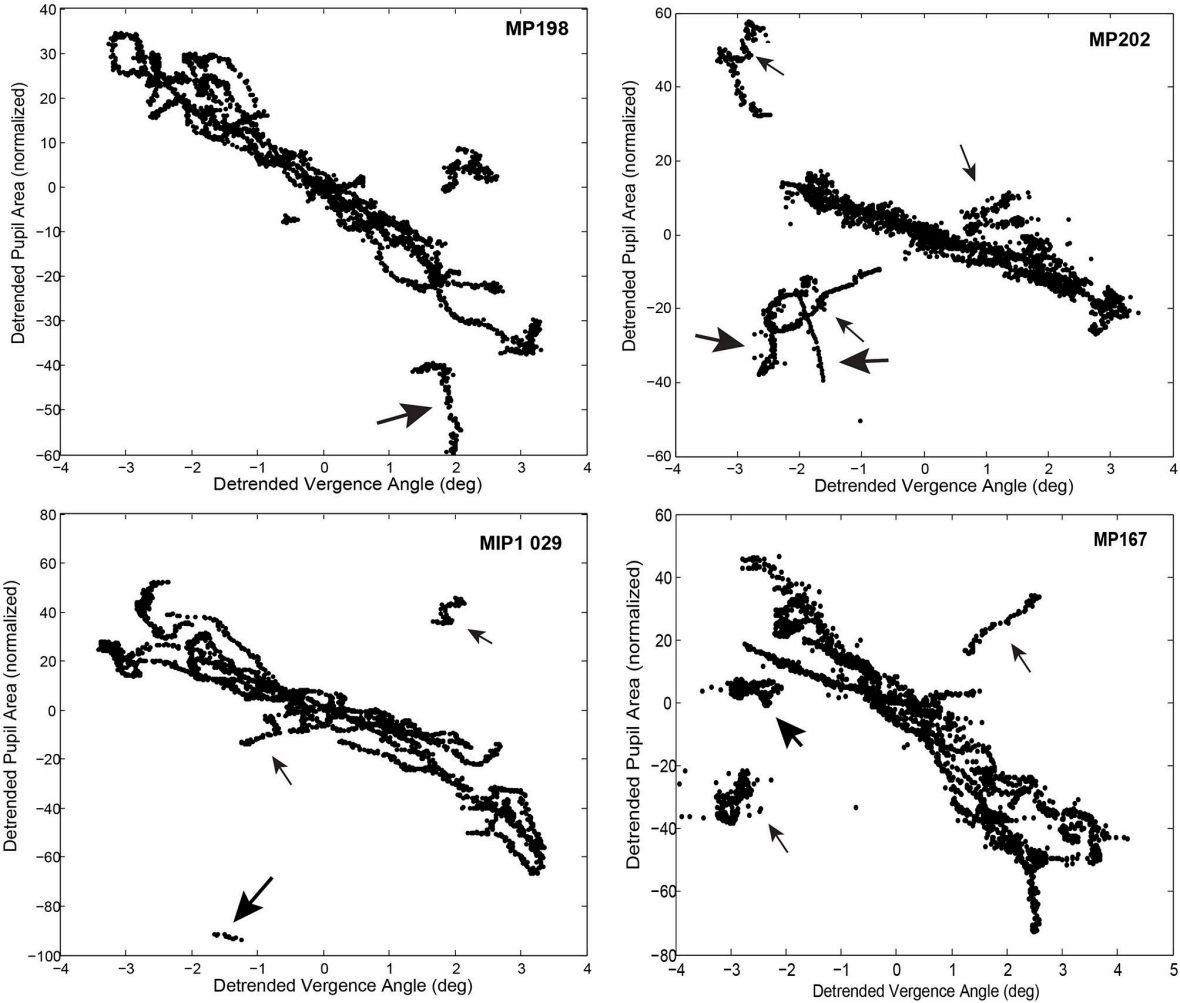
Time-implicit plots of detrended pupil area as function of detrended vergence angle, after subtraction of the pupil area intercept from each linear segment. Note the overlap between the segments representing a coordinated near response pattern. Thin arrows indicate segments with a positively correlated relationship between vergence angle and pupil area. Thick arrows indicate segments with little covariation (uncorrelated eye movement and pupil movement).

The consistency and dominance of the inverse linear relationship between pupillary area and vergence angle (“near response pattern”) is obvious after alignment of the segments by subtraction of the pupil-size intercept from each segment (Figure 8). The near response segments form a tight, overlapping cloud of points with a negative correlation, supporting the hypothesis that the parallel, negative slope segments in the raw data (Figure 4) are the products of a coordinated near response motor program with different pupil size set points. However, there were short periods when pupil area increased with ocular convergence (Figure 8, small arrows) and periods showing either vergence movements without pupil area changes or pupil area changes without eye movement (Figure 8, large arrows).

The linear segments that did not show the near response pattern were divided by the c-means cluster analysis (above) into segments with a positive slope (slope ≥2 % of pupil area per degree vergence, membership of at least 0.65 in the positive histogram mode in Figure 5) and flat response segments (absolute slope < 2 % of pupil area per degree vergence, membership < 0.65 in both modes). During the latter segments, the pupillary responses and eye movements are uncorrelated. During the former period, the positively sloped segments (213 total in 52 subjects; 4 segments per subject) averaged a slope of 15.5 ± 0.8 % of pupil area range per degree of vergence (mean ± SE), which was of similar magnitude, but of the opposite polarity to the near response, and a similarly robust within-segment *R*^2^ for the linear relationship (0.65 ± 0.02). Because the coefficient of determination was very strong during these epochs, it appears that binocular disparity is a prominent drive for pupillary dilation during convergence or pupillary constriction during divergence, which is opposite in polarity to the “near response.” However, the durations of these positive slope responses (1.17 ± 0.08 s, mean ± SE) were significantly shorter than the near response (negative slope) segments (Tukey HSD test, *p* < 0.01).

For the segments with a positive vergence angle-pupil size relationship, the vergence angle at the start of the segment accounted for ~54% of the variance in the (new) pupil size set point (Figure 6, lower panel and Table 3). The constant component was a dilation of only about 1% and the modulation was asymmetric. The set point for the pupil was more constricted during convergence with a peak modulation of 58% of normalized pupil size. The pupil set point was more dilated during divergence with a modulation of 38% of normalized pupillary range. When the pupil size and vergence angle traces from these “positive slope segments” are extracted and superimposed (right side of Figure 7), these asymmetric set point adjustments are reflected in discontinuities in the pupil response segments during convergence vs. divergence. Hence, these epochs seem to be a second motor program for pupillary regulation during binocular disparity-driven oculomotor responses.

**Table 3:** Estimated parameters for model components reflected in pupil intercept estimates for piecewise linear coordination patterns.

	**Offset**	**Linear slope**	**Modulation diverging (%)**	**Modulation converging (%)**	**Phase re: stimulus (dilation re: convergence)**
Near response pattern (*R*^2^ = 0.241)	3.96	−0.21 /s	14.51	14.75	0.50 rad lead (796 ms lead)
Positive-slope pattern (*R*^2^ = 0.543)	0.9	0.62 /s	30.88	58.18	2.98 rad lag

The flat response segments were infrequent observations (44/780 total segments in 52 subjects, ~1 segment per subject). Their slope was nearly zero (0.1 ± 1.8 % of pupil area range per degree of vergence), their durations (1.38 ± 0.19 s, mean ± SE) were comparable to the positive slope segments, and their within-segment *R*^2^ for the linear relationship (0.42 ± 0.05) was significantly lower than for the positive or negative slope segments (Tukey HSD tests, *p* < 0.01). These findings indicate that pupillary activity is uncorrelated with vergence during a small period of time during the binocular disparity tracking task.

**Figure 9:**
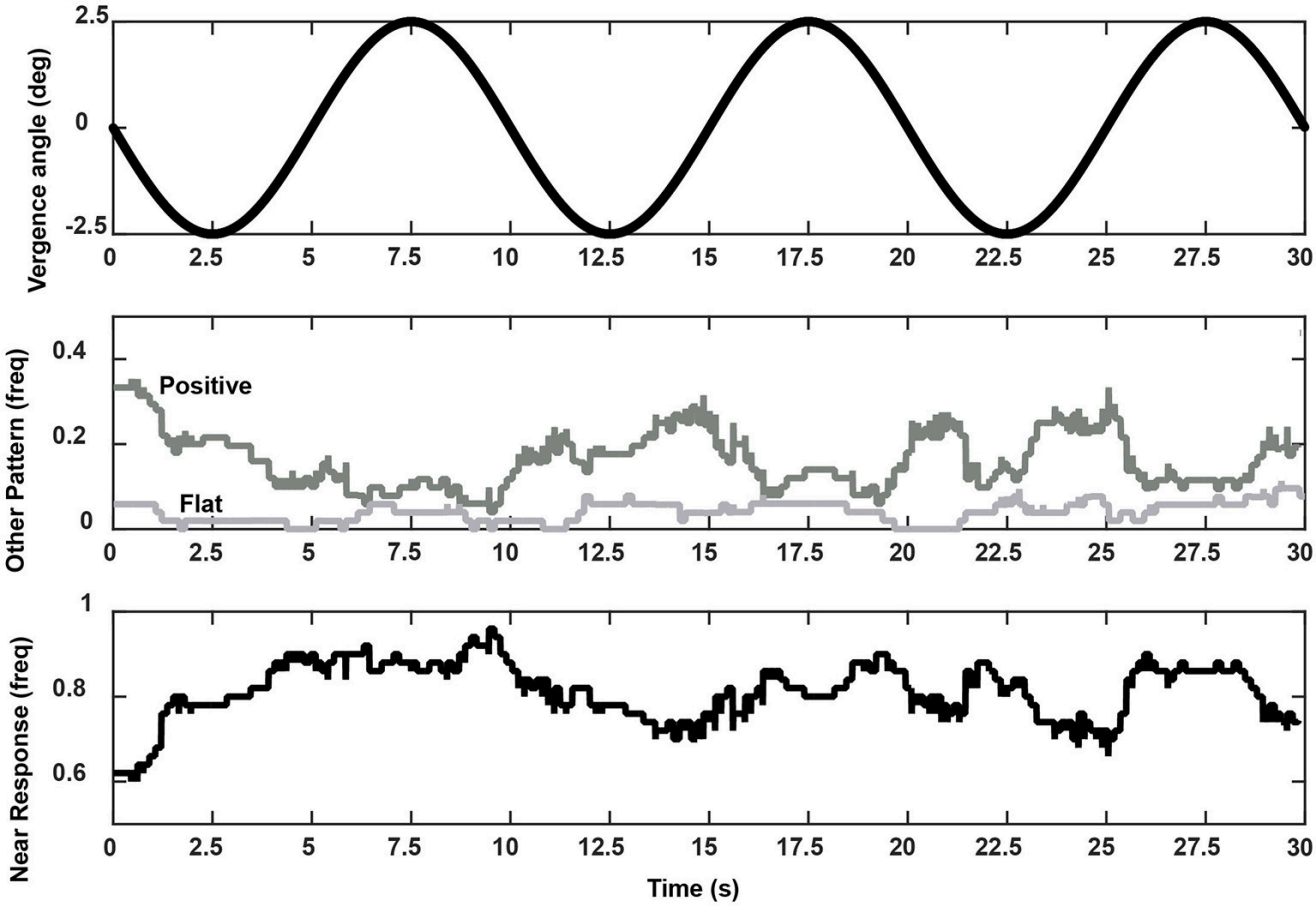
Frequency of occurrence of a coordinated near response segment (i.e., pupil constriction-convergence/pupil dilation-divergence linear relationship, lower panel), positive slope segment (i.e., pupil dilation-convergence/pupil constriction-divergence linear relationship), or flat relationship (middle panel) during the vergence task. These summaries are taken from pooled data from all 52 subjects. The upper trace shows the stimulus pattern (divergence negative re: baseline tropia).

The relative prevalence of linear segments showing a near response pattern and other pupil-oculomotor (flat or positive correlation) patterns during disparity vergence tracking are shown at each time point in Figure 9. Data from the single subject with low fidelity disparity vergence tracking are included. The vergence eye movements did not vary during the segments showing pupillary near responses or other responses; rather the differences in slopes can be attributed to the pupillary size responses alone (Figure 7). The near response pattern was most prevalent when the eye positions were converged (i.e., binocular disparity closer) relative to the initial tropia. The response segments showing the opposite relationship (e.g., constriction with divergence) were most common with divergence from the initial tropia point. The flat segments were uncommon and showed no preference for binocular disparity.

**Figure 10:**
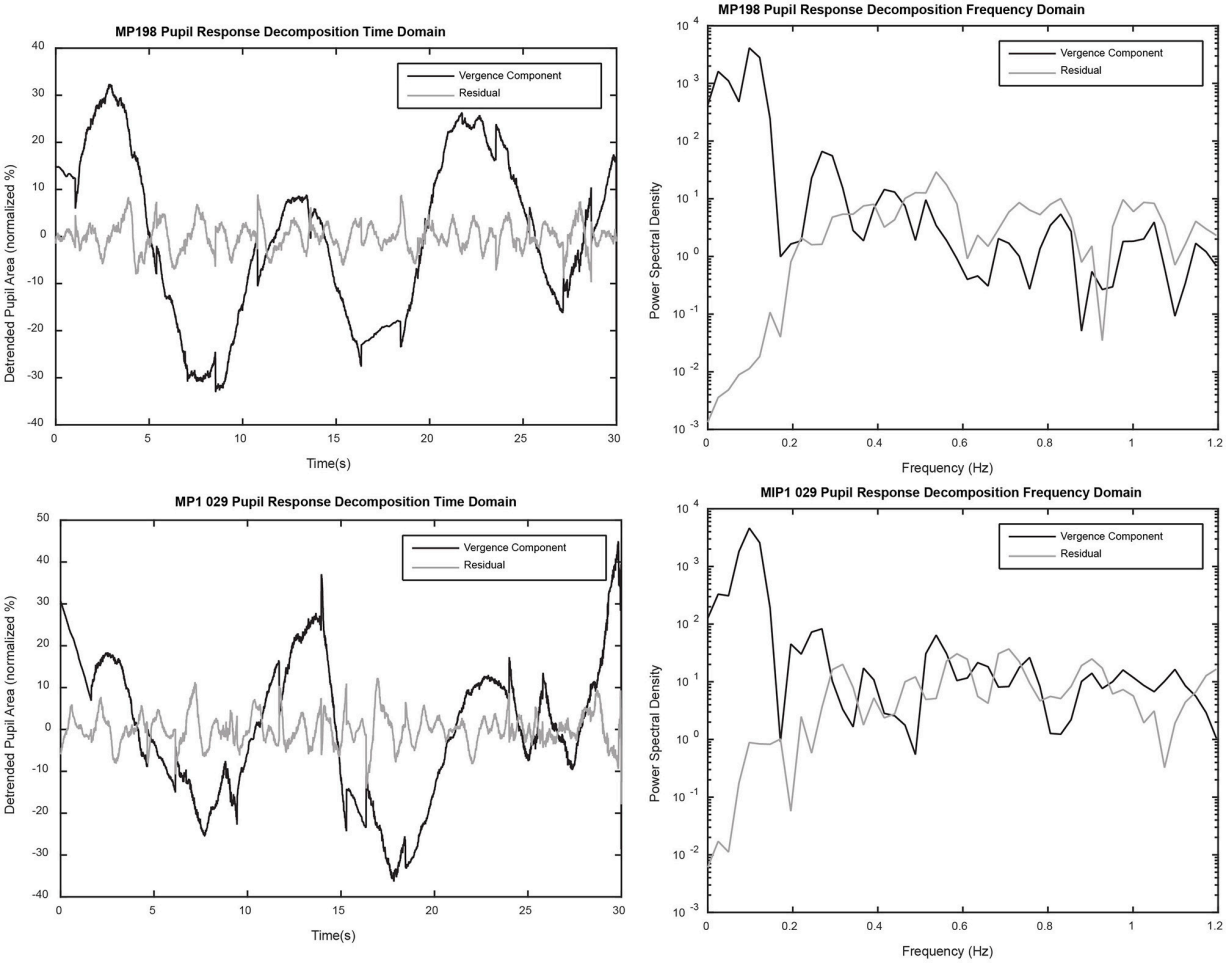
The left panels show a decomposition of the detrended pupil data into components predicted by (1) the relationship with detrended vergence eye movements alone (black) and (2) the residual (gray). The power spectral densities for each component (right panels) were calculated with the “periodogram.m” function in MATLAB (MathWorks, Inc., Natick, MA), using a full length Hamming window and a 4,096 point fast Fourier transform.

The linear segment analysis can also be used to decompose the pupil data into two components, a purely vergence eye movement related component (product of slope of linear segment and detrended vergence eye movement data) and the residual pupillary activity representing a component unrelated to vergence. The decompositions from two representative data sets are shown in Figure 10 (left panels). A frequency domain assessment of the relative contributions of these mechanisms to periodic changes in pupil area is shown in the right panels of Figure 10. Several features are notable from this analysis. First, activity coordinated with the 0.1 Hz disparity-driven vergence eye tracking is dominant at low frequencies (below ~0.25 Hz). Second, power spectral density was prominent in the hippus-like frequency range (0.5–0.7 Hz) in the residual data trace. Third, the appreciable power in the hippus frequency range (0.5–0.7 Hz range) of the vergence-associated pupil activity represents coordinated disparity-driven vergence-pupil responses.

#### Conjugate (versional) smooth pursuit task

**Figure 11:**
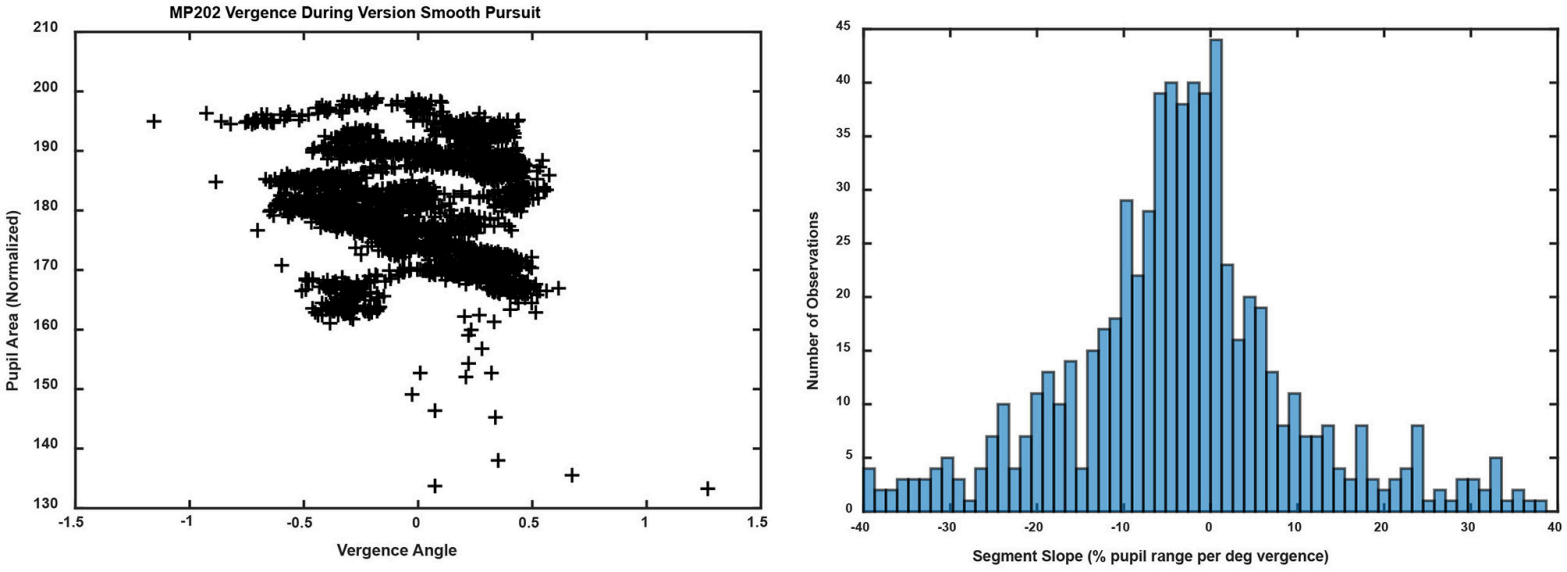
The left panel shows the trajectory of the vergence angle and pupil area for the same unit shown in Figure 5 (left panel). Note that the graph appears as a series of noisy quasi-linear segments, offset by differences in a pupil size set-point. The right panel shows the unimodal distribution of linear segment slopes.

In contrast to the dynamic binocular disparity pursuit task, there was no evidence of coordination of eye movements (conjugate or disconjugate components) and pupil size regulation during performance of horizontal or vertical conjugate smooth pursuit tasks (±10°) at 0.1 Hz. The versional movements were accompanied by variable, small vergence movements that were accompanied by pupil size changes. The macroscale analysis model had a relatively poor fit to the pupil data as a function of vergence movements, accounting for < 25% of the variance in the pupil size shifts [*R*^2^ = 0.232 ± 0.023 (S.E.,) compared to *R*^2^ = 0.51 ± 0.03 (S.E.) for the disparity pursuit task]. Application of the Gath-Geva algorithm identified linear segments of ocular vergence-pupil coordination with a lower *R*^2^ value (0.400 ± 0.012 S.E.) than the vergence pursuit task (0.655 ± 0.027 S.E.), which can be seen in the “noisy” trajectory of the pupil as a function of vergence eye position in Figure 11 (left panel, compare with Figure 5, left pane). The distribution of the slopes of the segments had a single peak (Figure 11, right panel). In terms of the divisions for the relationships during binocular fusion, 55.9% of the segments had negative slope (< −2% pupil area range per degree vergence), with a mean slope of −13.63± 0.65 (S.E.) pupil area range per degree vergence, an average duration of 1.86± 0.09 (S.E.) seconds and an average *R*^2^ value of 0.414 ± 0.016 (S.E.). The positive slope segments (>2% pupil area range per degree vergence) constituted 27.1% of the sample, had a mean slope of 17.68± 1.32 (S.E.) % pupil area range per degree vergence, an average duration of 1.71± 0.17 (S.E.) seconds and an average *R*^2^ value of 0.427 ± 0.023 (S.E.). Finally, the flat slope segments (between −2 and 2% pupil area range per degree vergence) constituted 17.0% of the sample, had a mean slope of −0.07± 0.10 (S.E.) % pupil area range per degree vergence, an average duration of 2.81± 0.19 (S.E.) seconds and an average *R*^2^ value of 0.353 ± 0.027 (S.E.).

## Discussion

This study utilized a head-mounted virtual reality display with integrated clinical eye tracking capabilities to characterize the association between pupil activity and vergence eye movements that are generated in response to a rapid (binocular disparity step) or a gradual (sinusoidal disparity pursuit) shift in binocular disparity of small targets. Because target luminance, sharpness and size were maintained, responses were purely to binocular disparity. They did not appear during conjugate pursuit on the same device. Robust vergence eye movements were elicited by convergent and divergent motion of fixation points presented to each eye. For a disparity fusion task, the disparity between target displays to each eye shifted abruptly to require movements (convergence or divergence) to resolve diplopia. For a disparity vergence pursuit task, disparity followed a sinusoidal profile with a period of 10 s. This approach is analogous to Rashbass and Westheimer's classic studies of disjunctive eye movements (23), which used cathode ray tubes for simultaneous monocular stimuli. As in the earlier studies for higher disparity frequencies, the gradual binocular disparity was sufficient to elicit sinusoidal tracking and concurrent pupil size changes, without changing either global luminance or apparent size of the fixation points.

In terms of the model in Figure 1, the stimulus-related eye and pupil movements during binocular disparity stimulation are a function of (a) the eye movement vergence response to resolve the disparity and (b) the response dynamics of the pupil to the internal signal driving the vergence eye movements. The coordination between vergence eye movements and pupil area was analyzed from two perspectives, which we can term macroscale and microscale. The macroscale analysis analyzed the responses as a single continuous process, which estimates parameters for vergence eye movements, pupil area changes, and coordination of the eye and pupil movements with an implicit assumption that pupil size at zero vergence remains invariant. The microscale analysis assesses the movements as a sequence of discrete epochs of coordinated activity, which includes explicit identification of (a) recalibrations of the pupil area at zero vergence and (b) epochs of synkinetic activity with different operating characteristics for pupil area adjustments in relation to vergence eye movements.

The combination of a disparity step test and a disparity smooth pursuit task at a single, low frequency permit an examination of synkinetic control in a ballistic (step) versus a pursuit task. For the ballistic movement task, the model analysis suggested that 28 ± 3% of the variance of the pupil response could be explained by modeled coordination with the vergence eye movements alone. For the pursuit task, a larger proportion (51 ± 3%) of the variance of the pupil response was explained by the pupil movements modeled from the vergence behavior. Further, the pupil sensitivity for the pursuit task (−8.13 ± 0.75 % area/° convergence) was significantly greater than for the step task (−6.51 ± 0.56 % area/° convergence). The residual activity (unrelated to dynamic ocular convergence) includes physiological hippus (15), slower pupillary fluctuations related to respiratory cycle control and/or respiratory sinus arrhythmia (16), attentional load and task experience (17). These findings motivate a more detailed study of the frequency dependence of contributions by disparity resolution control to pupil movement.

Analyses indicated that there is an active recalibration process for baseline pupil area during both binocular disparity tasks, which sets a control point for pupil aperture (area). This is obvious from stimulus cycle-to-cycle shifts of the pupil size at zero vergence in bivariate plots of pupil area as a function of the vergence angle (Figures 4, 5). For the disparity pursuit task, they varied weakly with eye position (Figure 6). The relationship between pupil area and vergence angle during a binocular disparity pursuit task also displayed epochs with two different patterns for coordinated pupil and vergence eye movement control, as well as infrequent epochs of independent regulation of eye movements and pupil area. The eye movement and pupil area measurements were coordinated closely more than 90% of the time during this binocular disparity-induced tracking task. These movements could be divided analytically into epochs of (1) “near response” (linear negative relationship between pupil area and vergence angle [convergence positive]), (2) positively correlated response (linear positive relationship between pupil area and vergence angle), and (3) uncorrelated response epochs. These relationships dominate bivariate plots of the instantaneous pupil size as a function of instantaneous vergence angle (e.g., Figures 5, 8). The tight linear correlation (average *R*^2^ of at least 0.65) for the two former response epochs suggest that they represent distinct control modes for pupillary control during visual tracking of approaching and receding targets, governed by opposite polarities of drive from the binocular disparity control mechanism postulated in the literature (1). There are also different behavior patterns governing the pupil set-points for the linear trajectories for both the “near response” segments and the positive correlation segments, suggesting that they represent different polarity pupil area control programs during vergence tracking. Epochs of these pupillary control modes are also recognizable in the binocular disparity fusion task responses (Figure 4). Because the vergence eye movement responses followed stimuli with extremely high fidelity, we suggest that the apparent coordination between disparity-driven ocular and pupillary responses is produced by a real-time selection of different modes of disparity controller drives for pupillary control. This effect is unrelated to hippus, which remains when the vergence eye movement-dependent activity is subtracted from the pupil data (Figure 10, left panels).

**Figure 12:**
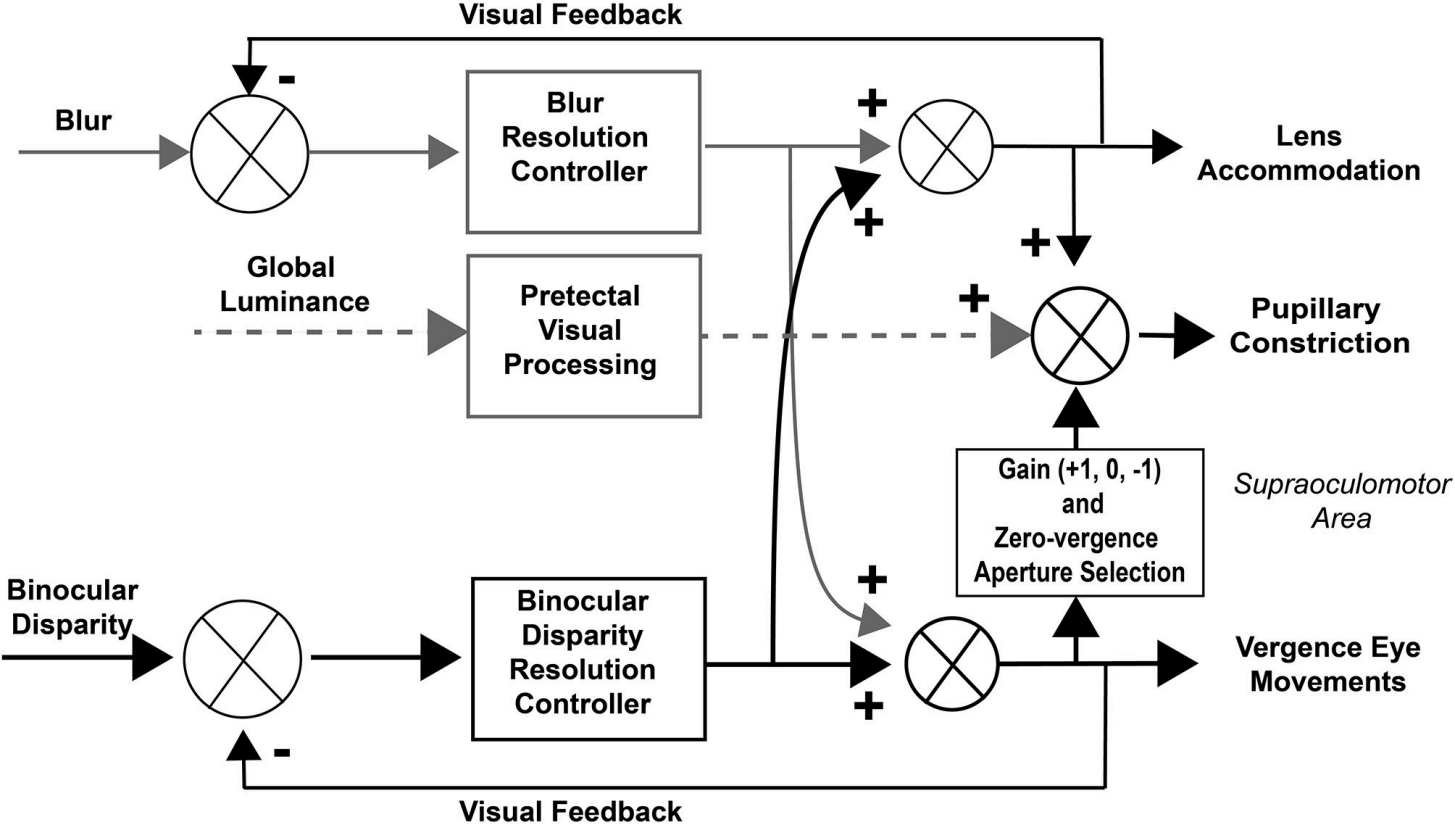
Modified dual interaction model. This model adds a three-state gain selection switch and zero-vergence aperture reset for the influence of disparity signals on pupillary control to the basic McDoughal and Gamlin model (1), which accounts for the epochs of pupil-vergence synkinesia that are observed during 0.1 Hz binocular disparity tracking. The binocular disparity pathways are highlighted in black to mirror the selective stimulation used in this study.

The consistency and fidelity of the vergence eye movements support the view from previous studies that they reflect the output of a binocular disparity controller, which eliminates diplopia by moving the eyes to fuse the disparate stimulus features (1, 3). Previous studies have then viewed the pupillary, vergence, and lens accommodation during near responses as the result of interactions between binocular disparity and blur controllers that produce a continuous, coordinated pupillary constriction with ocular convergence and pupillary dilation with ocular divergence (1, 3). The results of this study suggest the former approach is inadequate for explaining dynamic properties of responses driven by binocular disparity in isolation. Rather, the eye movement and pupillary responses during resolution of either a step or a sinusoidal binocular disparity stimulus appear to consist of successive epochs of uncorrelated activity, coordinated near response activity (pupil constriction with convergence/dilation with divergence) and a coordinated opposite response pattern (pupillary dilation with convergence/constriction with divergence). The piecewise linearity of pupil area regulation with respect to vergence angle suggests that the controller co-regulates iris dilator and sphincter muscle activity. It seems premature to propose an alternate model from these unexpected findings. However, one may speculate (Figure 12) that program selection may be mediated by cerebro-ponto-cerebellar networks influencing premotor mechanisms in the supraoculomotor area (1, 18).

In a study of workers with prolonged near vision work at video displays, Ukai et al. (24) reported an adaptive increase in pupillary constriction at 0D accommodation relative to the prolonged near vision work period. Because the depth of field of the human eye varies as the reciprocal of pupil diameter (25), the modulation in the set points for pupil area regulation may be setting tolerances for blur during disparity-driven tracking. If we assume an average pupil diameter dynamic range of 2.5–8.5 mm, then the pupil area dynamic range will vary between 4.9 and 56.7 mm^2^ and the midpoint of the area range (zero on the detrended normalized plots) will be 30.8 mm^2^ (diameter of 6.3 mm). Hence, 10% of the range would be 5.2 mm^2^. For the near response pupillary control epochs, the modulation of the pupil area operating point for each segment relative to initial (calibration baseline) tropia (defined as zero vergence) is relatively small (±15% pupil constriction re: 50% area during divergence) and noisy (*R*^2^ = 0.24). By contrast, after the selection of a positive relationship between pupil area and vergence angle, the set points for pupil size regulation are more tightly correlated, with vergence angle relative to the initial tropia (*R*^2^ = 0.54), and the magnitudes are greater. The modulation magnitudes are equivalent to ~−5.2 mm^2^ (constriction re: 50% area) during convergence and +1.7 mm^2^ (dilation re: 50% area) during divergence. These findings suggest collectively that the near response segments and the positively correlated segments are pupillary motor programs to produce different depth of field attributes during the performance of vergence eye movements. The different epochs of correlated eye movement and pupil size activity were associated preferentially with convergent vs. divergent eye alignment. The near response segments were most prevalent when the eye alignment was convergent (relative to initial or baseline tropia), while the converse was true for the segments showing a positive correlation between pupil area and vergence angle. The segments showing flat (no correlated) relationship were infrequent and showed no clear preference for eye alignment.

The effects of variable iris diaphragm apertures on image properties are well known to expert photographers. Techniques for image *bokeh* provide insight into the utility of different motor programs for regulating both the set point and dynamic control of pupil area during disconjugate eye movements. In photography, bokeh is achieved by opening the iris diaphragm to reduce the depth of field, which produces a subtle blur of objects that are not precisely within the focal plane. This strategy establishes a subtle demarcation of figure (target) from background relationships (26). From this aperture control perspective, two features are apparent from the piecewise linear relationships between pupil area and ocular vergence. Firstly, the resetting of the “baseline” pupil size at zero vergence for each segment may set a static baseline blur of the background relative to the target for a linear variation of pupil area with vergence angle. Secondly, we observed two different scenarios for dynamic aperture effects during disparity-driven vergence. A first scenario is dilation of the pupil during divergence (a component of the “near response”). The second scenario is dilation during convergence, which is a component of the coordinated response with opposite polarity, that occurs preferentially at diverged disparity targets (about 30% of the time). Ueda et al. (27) reported that the threshold velocity for dynamic visual acuity (Landot C orientation at constant distance from subject) increased significantly in subjects after mydriasis induction with Mydrin-P (phenylephrine) eye drops. Because topical phenylephrine does not affect lens accommodation (28), the change in dynamic acuity suggests that the dilating aperture effects may increase the dynamic visual acuity range for tracking target motion when binocular disparity fusion requires divergence of the eyes.

Conversely, the dominant pattern of dynamic pupillary constriction during convergence (near response) increases depth of field and decreases blur as the eyes converge. An opposite scenario was episodic pupillary constriction with divergence, particularly when the targets were diverged from the center (positive slope pattern). From a perceptual perspective, this strategy would decrease blur and facilitate target identification. In addition, the increased depth of field would facilitate alignment of the right and left disparate targets in the face of a background. In summary, co-regulation of convergence and pupil area can be considered as control system that is useful in setting figure (target) to background relationships during dynamic tracking, with concomitant generation of blur to affect control of lens accommodation (Figure 1). Rather than a simple reflex, the multiple patterns of coordination suggest that we view this circuit as an interactive controller that sets an aperture effect characteristic for epochs of visual information sampling. Hence, it will be of considerable interest to investigate the occurrence of the repertoire of dynamic pupillary control patterns under viewing conditions that include blur and relative size changes of the targets.

## Ethics statement

This study was carried out in compliance with human subject ethical guidelines and regulations of United States government agencies, including Departments of Health and Human Services and the Food and Drug Administration. The protocol was approved by the Institutional Review Boards of the University of Miami (Protocol #20150286), Madigan Army Medical Center (#214005, IRBNet #393240-1) and Naval Medical Center, San Diego (#NMCSD.2013.060). All subjects gave written informed consent in accordance with the Declaration of Helsinki.

## Author contributions

CB and MH contributed to design, data analysis, and writing the manuscript. MS contributed to study coordination, data collection, and writing the manuscript. AK and RA contributed to technical aspect of hardware and device software design, both for implementation and in the methods section of the manuscript.

### Conflict of interest statement

AK and RA are employees of Neuro Kinetics, Inc. The views expressed herein do not necessarily reflect the official policy or position of the Department of the Navy, the Department of the Army, Department of Defense or the U.S. Government. The roles of AK and RA were limited to device design and technical aspects of manuscript preparation. Hence, their employment by Neuro Kinetics, Inc. has no influence on the analysis, discussions, or conclusions of this manuscript. The remaining authors declare that the research was conducted in the absence of any commercial or financial relationships that could be construed as a potential conflict of interest.

## Supplementary material

Figure S1 These graphs show the appearance of eye tracking data for subject MP198 during the binocular disparity pursuit task. The detrended data are shown in Figure 2B.
