# Supplementary figures and images for: Patterns of Pupillary Activity During Binocular Disparity Resolution
Source: Front Neurol. 2018 Nov 26;9:990. doi: 10.3389/fneur.2018.00990 (PMC6276540; doi:10.3389/fneur.2018.00990)

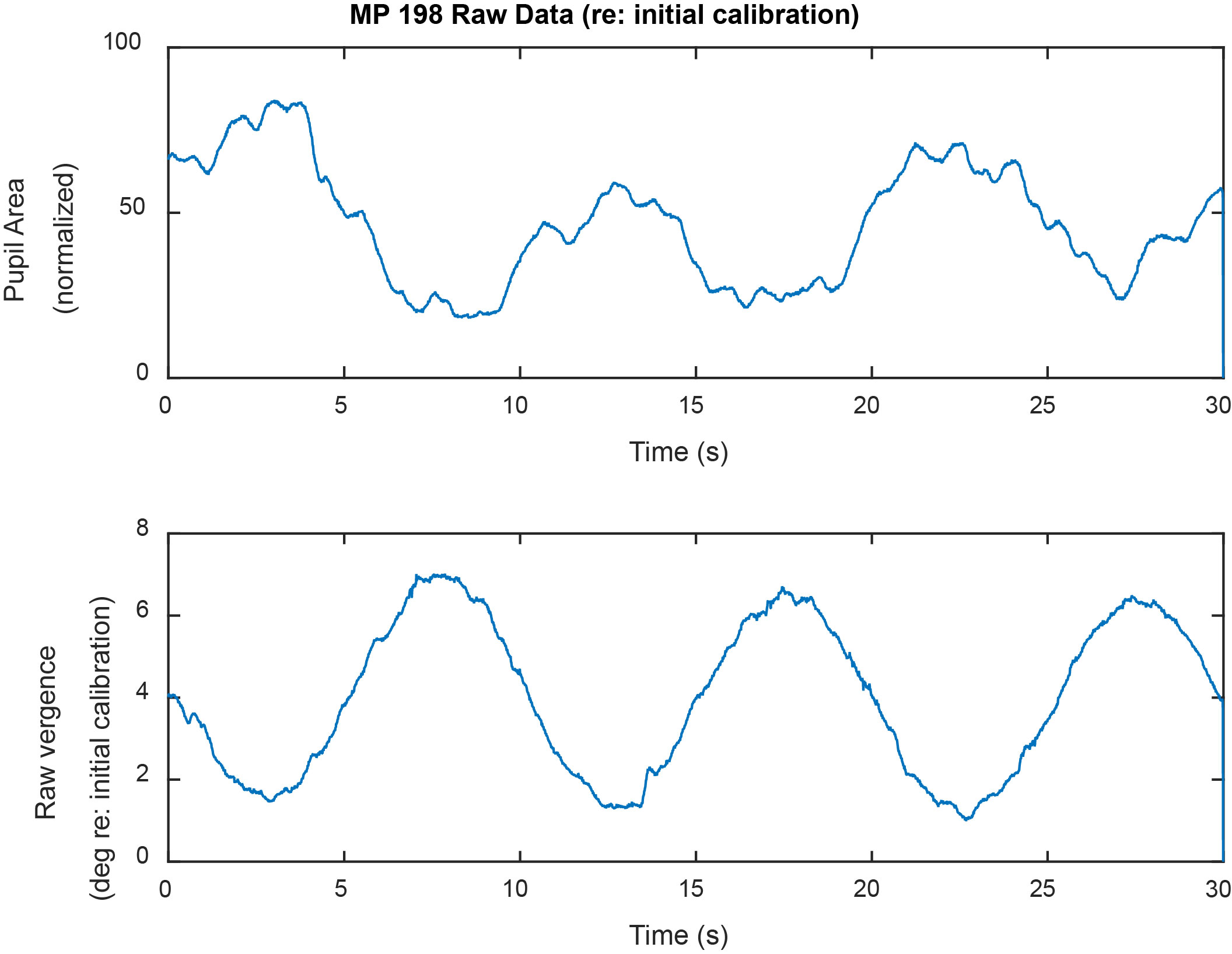

Supplement: Figure S1 — These graphs show the appearance of eye tracking data for subject MP198 during the binocular disparity pursuit task. The detrended data are shown in Figure 2B. [file Image_1.JPEG]
